# 2566 Personalized models of distal airway epithelial-stromal unit in COPD

**DOI:** 10.1017/cts.2018.106

**Published:** 2018-11-21

**Authors:** Seyed B. Mahjour, Kazunori Gomi, Busub Lee, Olivier Elemento, Scott Randell, Renat Shaykhiev

**Affiliations:** 1 Department of Medicine, Weill Cornell Medical College, New York, NY, USA; 2 Department of Physiology and Biophysics, Weill Cornell Medical College, New York, NY, USA; 3 Marsico Lung Institute/Cystic Fibrosis Center, The University of North Carolina at Chapel Hill, Chapel Hill, NC, USA

## Abstract

OBJECTIVES/SPECIFIC AIMS: The objective of this study is to develop patient-derived “personalized” organotypic models of human distal airways, in which basal stem cells (BCs) isolated from the pre-/terminal conducting airway region are co-cultured with autologous stromal cells from the same region to reproduce patient-specific distal airway epithelial-stromal units and their remodeling in COPD. METHODS/STUDY POPULATION: We established a protocol to isolate and propagate epithelial BCs, fibroblasts, and endothelial cells from the distal airways of normal and COPD lung donors. Heterogeneous cellular and molecular phenotypes in the human distal airways were characterized using immunofluorescence and single-cell RNA sequencing. Patient-specific distal airway epithelial-stromal units were reconstructed by co-culturing BCs and autologous stromal cells using an air-liquid interface-based airway wall model and a bronchosphere-based 3D distal airway organoid assay. RESULTS/ANTICIPATED RESULTS: Histologic analysis of derived epithelial-stromal units revealed heterogeneous patient-specific phenotypes characterized by hypo-/hyper-/metaplastic lesions (hypo-regenerative phenotype, mucous cell hyperplasia, squamous metaplasia, distal-to-proximal repatterning) in the epithelial compartment, accompanied, in some samples, by stromal remodeling. Candidate epithelial-stromal cross-talk mechanisms were identified using quantitative real-time RT-PCR analysis of autologous epithelial and stromal compartments of established patient-specific distal airway unit models. DISCUSSION/SIGNIFICANCE OF IMPACT: Epithelial and stromal cells isolated from distal airways of subjects with and without COPD can be assembled into functional, organ-level tissue which mimics the architecture of human distal airways and, in patients with COPD, reproduces several distal airway remodeling phenotypes. Patient-specific models of distal airway epithelial-stromal cross-talk established in this study can be used to identify candidate pathways that mediate disease-relevant airway remodeling and potentially utilized as pre-clinical platforms for developing personalized therapeutic approaches to suppress the progression of distal airway remodeling in chronic lung diseases, including COPD.

